# Supplementary material for: Hijacking Sexual Immuno-Privilege in GBM—An Immuno-Evasion Strategy
Source: Int J Mol Sci. 2021 Oct 12;22(20):10983. doi: 10.3390/ijms222010983 (PMC8536168; doi:10.3390/ijms222010983)
Supplement: Supplementary file 1 [file ijms-22-10983-s001.zip › ijms-1369321-supplementary.pdf]

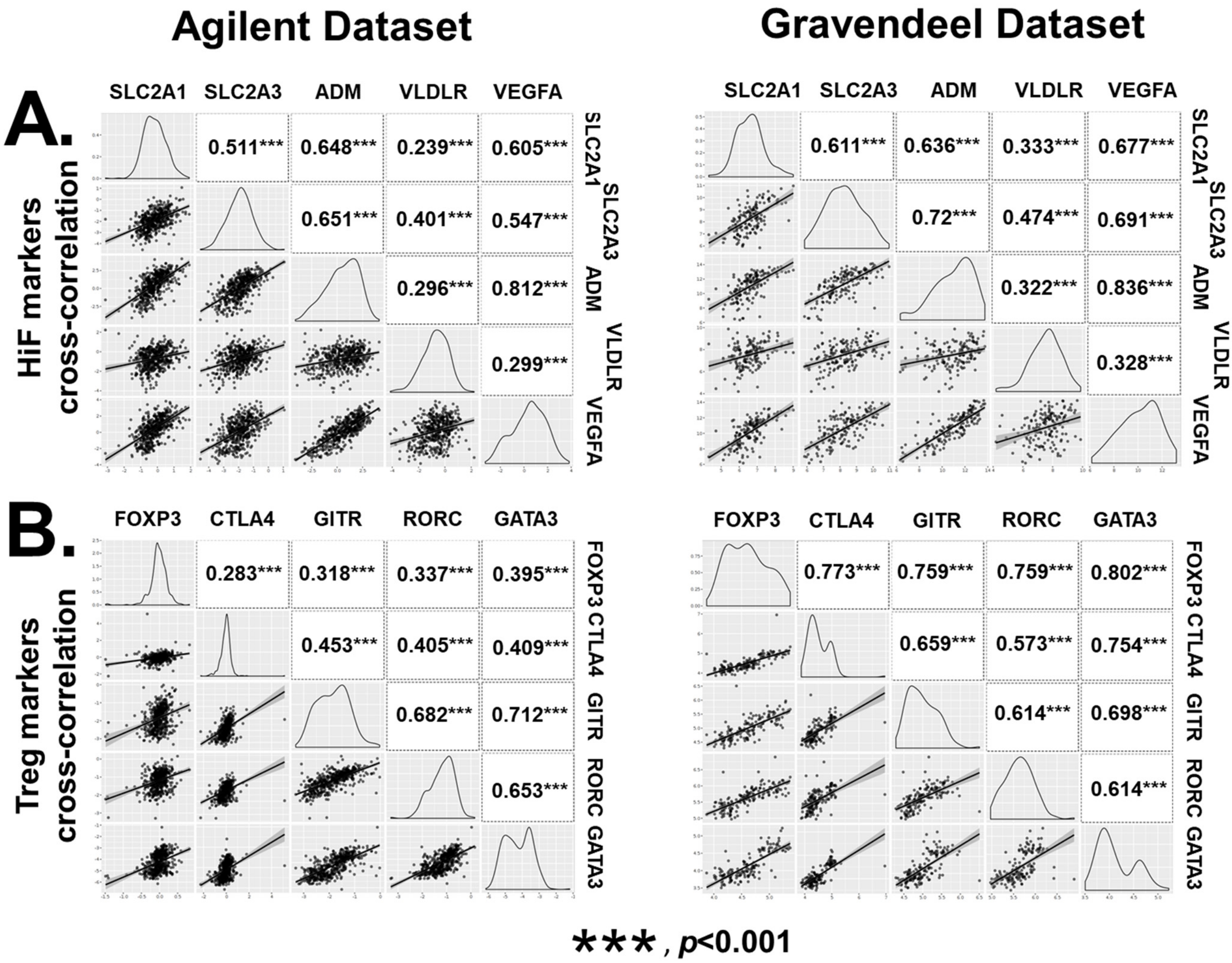

Figure S1. Cross-Correlation of Selected Hypoxia Response Genes (HiF) and RORC-Treg Genes in GBM: The cross-correlations of *SLC2A1*, *SLC2A3*, *ADM*, *VLDLR* and *VEGFA* (**Figure S1A.**) with *FOXP3*, *CTLA4*, *GITR*, *RORC* and *GATA3* (**Figure S1B.**) Transcription levels of wild-type IDH GBM tumors, drawn from Agilent, on the left, and Gravendeel, on the right, databases. $p<0.001$ in all cases. Images generated using Gliovis.

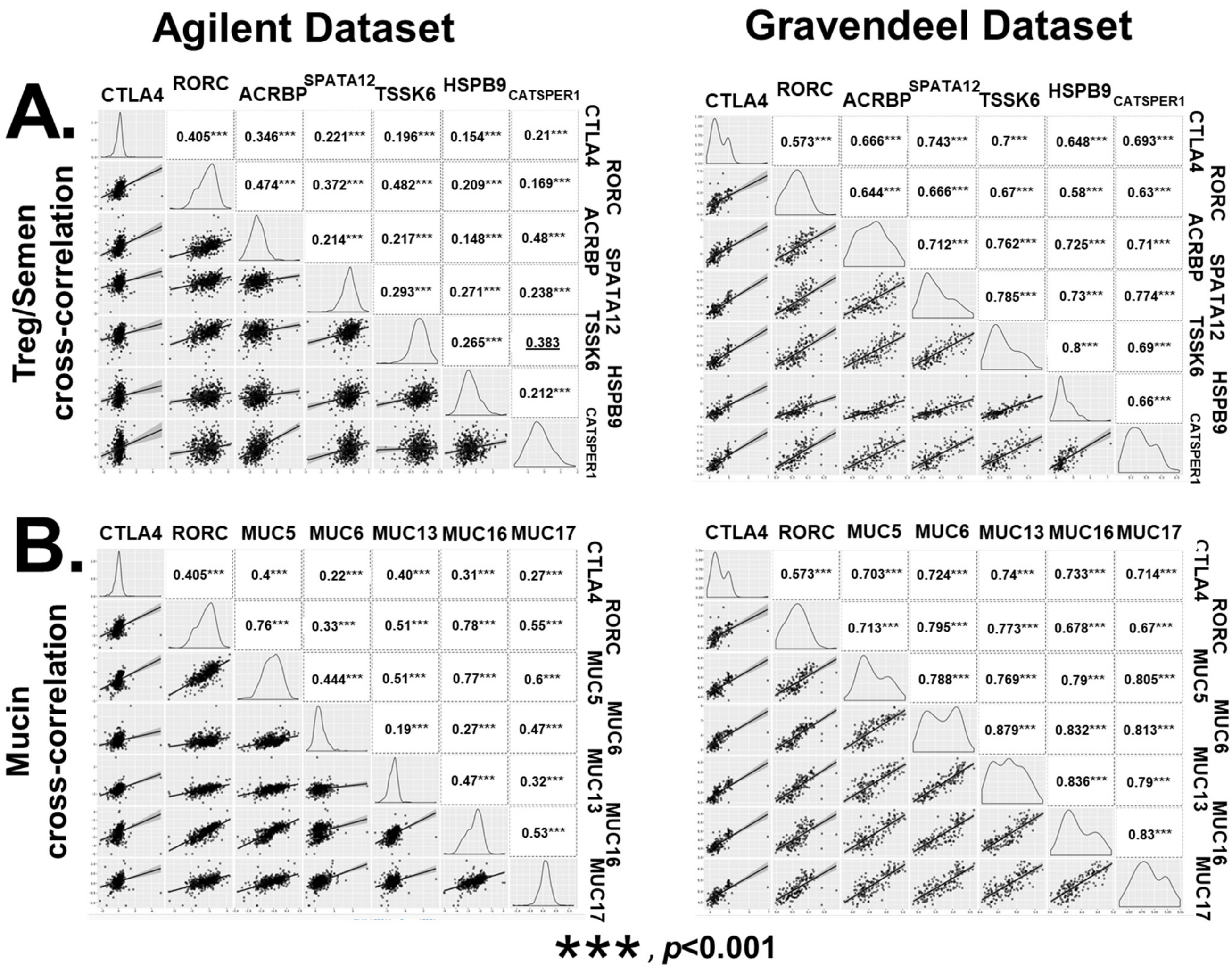

Figure S2. Cross-correlation of CTLA4 and RORC with male-specific and mucin genes in GBM: In Fig S2A the cross-correlations of *CTLA4* and *RORC* with the sperm specific genes *ACRBP*, *SPATA12*, *TSSK6*, *HSPB9*, and *CATSPER1*. In Fig S2B the cross-correlations of *CTLA4* and *RORC* with the mucin genes *MUC5B*, *MUC6*, *MUC13*, *MUC16* and *MUC17* are shown. These genes are native to both testicle and semenized cervix, both known to be rich with RORC-Tregs. The highly significant cross-correlations with *CTLA4* and *RORC* would suggest this RORC-Treg subtype is reconstituted in GBM. Transcription levels of wild-type IDH GBM tumors, drawn from Agilent, on the left, and Gravendeel, on the right, databases. $p<0.001$ in all cases. Images generated using Gliovis.

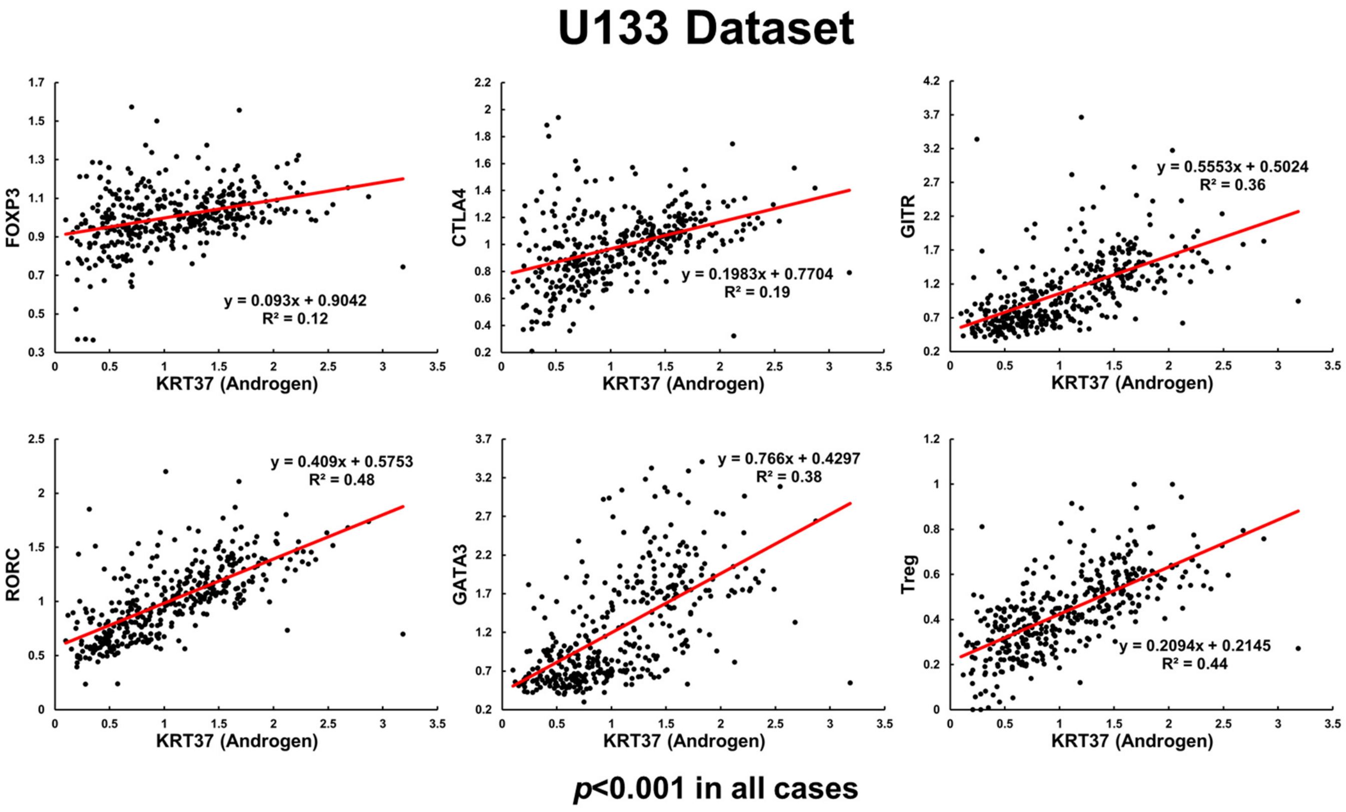

Figure S3. Cross-correlation of RORC-Treg genes with androgen report gene *KRT37*, in GBM: The cross-correlations of individual RORC-Treg markers and Tregs with androgen responsive gene *KRT37*. GBM tumors, wild-type IDH, were drawn from the U133 Firehose Legacy database; correlations are $p<0.001$ in all cases.

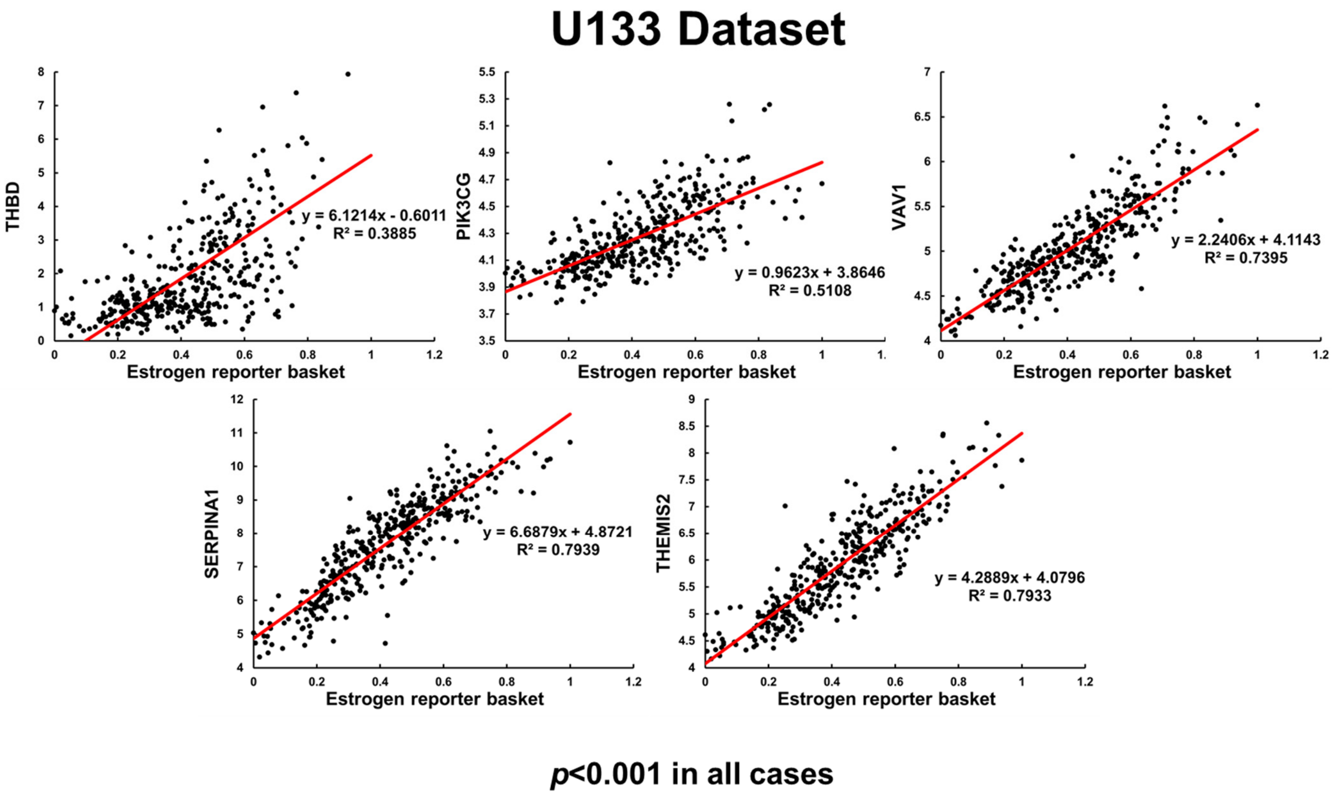

Figure S4. Cross-correlation of estrogen sensitive genes in GBM: The cross-correlations of GBM tumor mRNA levels of estrogen gene proxies with the calculated 'Estrogen reporter basket': derived by the averaged/normalized levels of the five estrogen reporter genes. Data for GBM transcriptome, wild-type IDH, were drawn from the U133 Firehose Legacy database and correlations are $p<0.001$ in all cases.

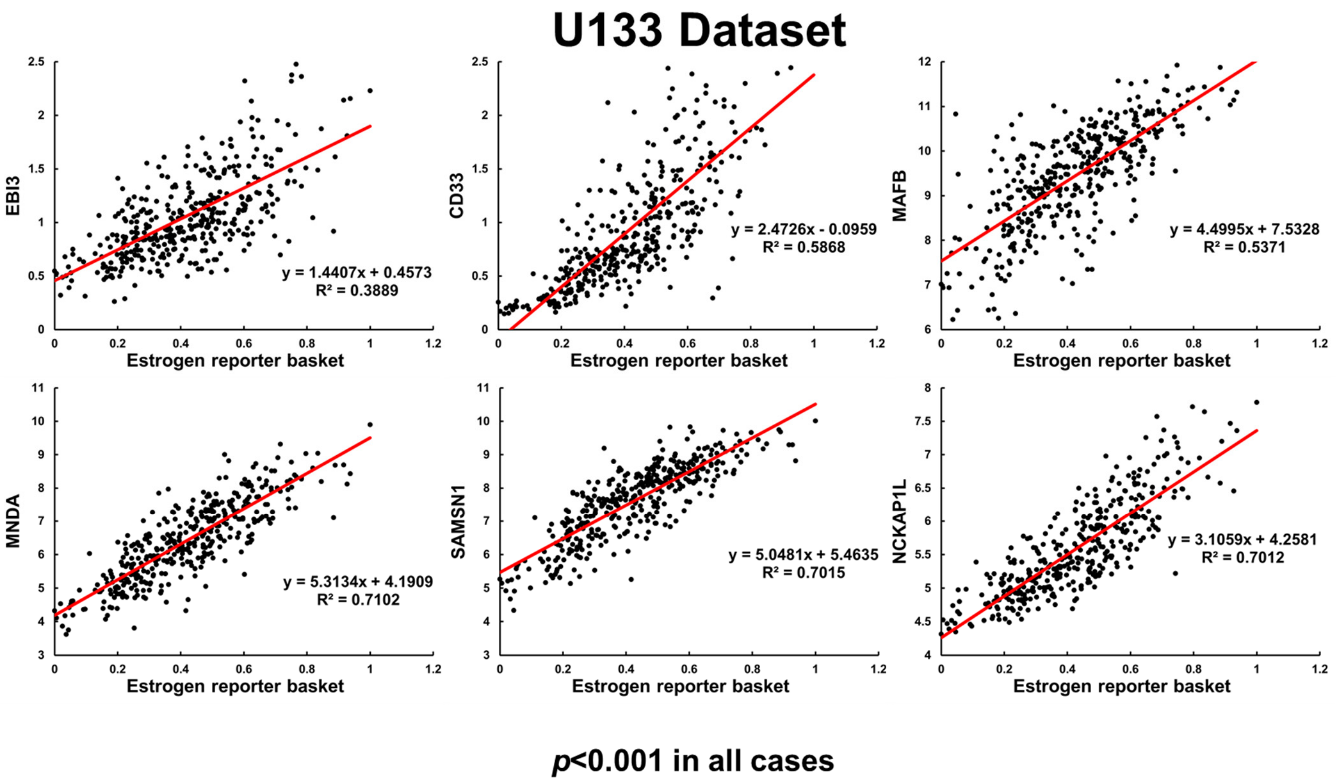

Figure S5. Cross-correlation of MDSC/TAM markers and estrogen reporter basket in GBM: The cross-correlations of MDSC and TAM gene markers with synthetic 'Estrogen' series derived from the averaged rescaled mRNA levels of estrogen responsive genes. GBM tumors, wild-type IDH, were drawn from the U133 Firehose Legacy database and correlations are *p*<0.001 in all cases. MDSC= myeloid derived suppressor cell; TAM= tumor associated macrophage.

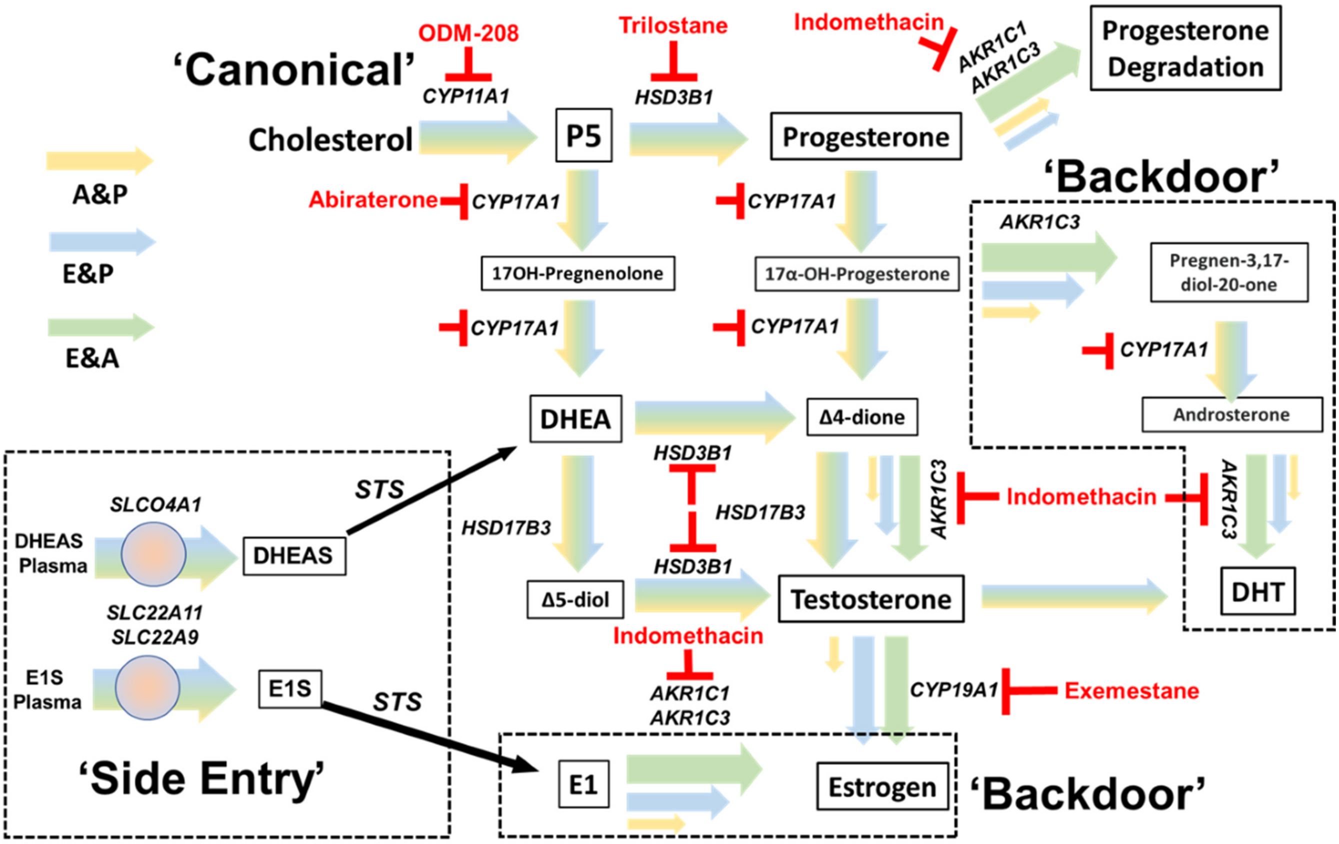

Figure S6. Steroid Synthesis Pathway with Enzyme Inhibitors: Depiction of classical, backdoor and side entry steroid synthesis pathways with inhibitors in red. Yellow denotes high androgen/progesterone; low estrogen (A&P), blue denotes high estrogen/progesterone; low androgen (E&P), and green denotes high estrogen/androgen; low progesterone (E&A). Arrow size correlates directly with level of upregulation. Tricoloured arrows designate equal upregulation among all three groups. **Canonical:** Cholesterol converts to P5 via CYP11A1, inhibitable by ODM-208. CYP17A1 converts P5 to 17OH-pregnenolone and then into DHEA, blockable by abiraterone. DHEA then converts into Δ5-diol by AKR1C3 or HSD17B3, and further converted to testosterone via HSD3B1, inhibitable by trilostane. DHEA can also be converted to Δ4-dione via HSD3B1, further converted to testosterone by AKR1C3 or HSD17B3, and proceeds to DHT via SRD5A or AKR1C3. **Side entry/Backdoor:** GBMs also use the fetal androgen synthesis pathway. The 'side entry' pathway starts with DHEAS and E1S with SLCO4A1, SLC22A11, SLC22A9 and STS, used to convert DHEAS to DHEA to Δ4-dione to testosterone, estrogen and DHT. The backdoor pathway starts with a diversion of the 17-α-OH-progesterone into pregnen-3,17-diol-20-one via AKR1C3. CYP17A1 generates androsterone which is converted into DHT by AKR1C3, bypassing the canonical testosterone route. This also converts estradiol to estrone via aldo-keto reductases 1 and 3, both inhibitable by indomethacin.

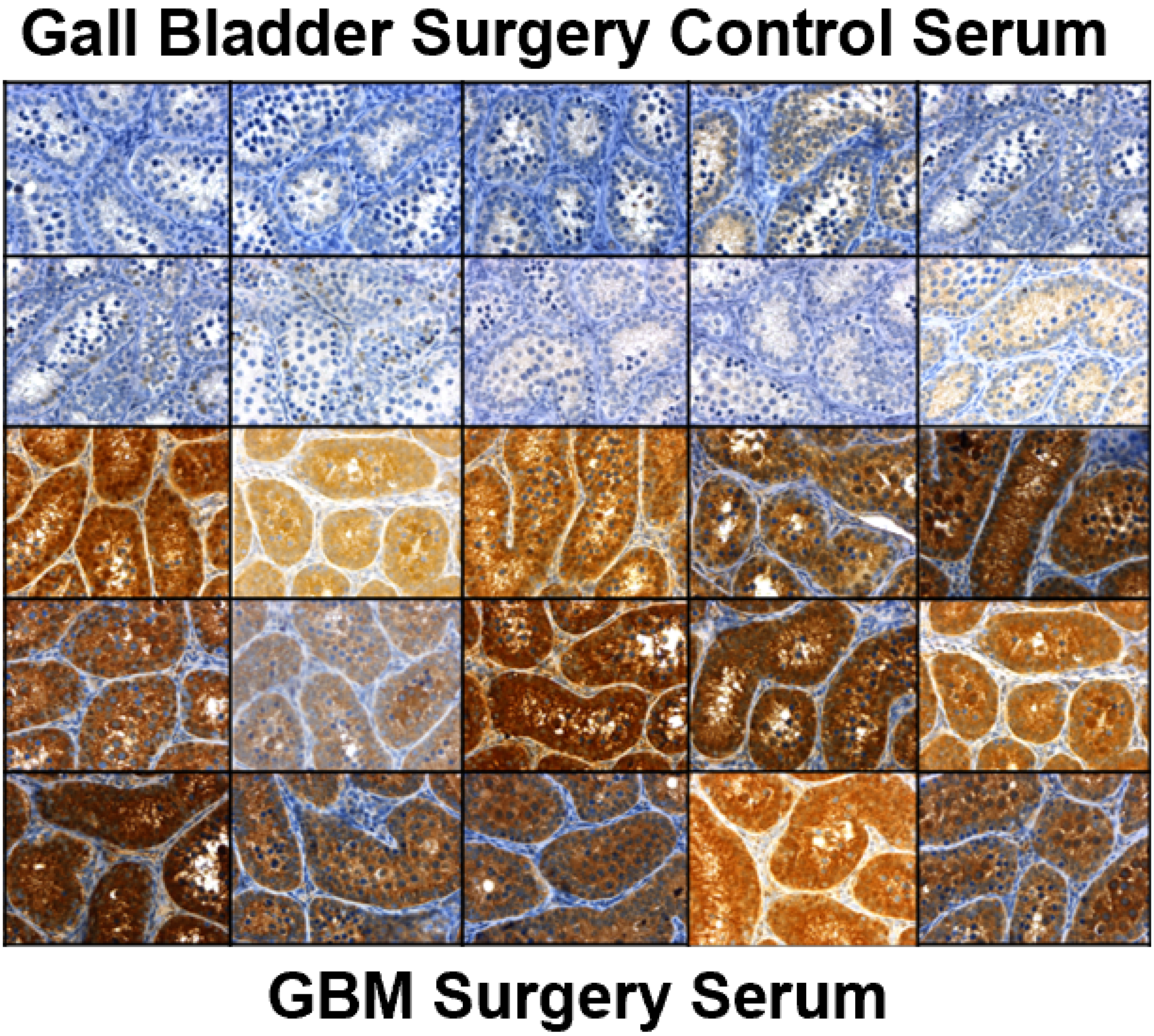

Figure S7. Monkey Testicle labeled with GBM and Control Patient Serum: Patients serum anti-testicular antibodies assayed using fixed cynomolgus monkey testicle slices. Top two rows consist of serum from control patients, showing low levels of anti-sperm/testicular antibodies, less brown staining. The next three rows are GBM patient serum, showing evidence of antibodies to testicular germ cells, seen deeply stained (brown).

Table S1. Characteristics of stratification of GBM tumors by HiF and Treg fractionation, we *p*=values derived from t-tests performed against the neither cohort.

U133 Dataset		Normalized enrichment of gene or gene basket														
Cohort	Pop %	SLC2A1	SLC2A3	VEGFA	VLDLR	ADM	HiF	FOXP3	CTLA4	GITR	RORC	GATA3	Treg	MG	MG Inflam	MG ↑↑NF-kB
A-Treg	28.5	1.21	1.77	0.85	1.07	1.10	1.08	1.17	1.35	2.70	1.82	3.32	2.08	0.69	1.66	0.86
B-Treg&HiF	21.6	1.91	2.91	2.80	1.78	1.48	2.20	1.12	1.34	2.67	1.69	2.77	1.90	0.84	1.81	2.03
C-HiF	23.9	1.70	2.24	2.91	2.09	1.39	2.09	1.00	0.97	0.93	0.96	0.95	0.98	1.12	1.21	1.16
D-Neither	26.0	1.0	1.0	1.0	1.0	1.0	1.0	1.0	1.0	1.0	1.0	1.0	1.0	1.0	1.0	1.0
Cohort		*p*=value of stratified group with respect to 'neither'														
A-Treg		>0.05	2E-04	>0.05	>0.05	>0.05	>0.05	5E-06	6E-14	5E-18	3E-19	1E-12	9E-21	1E-06	9E-04	>0.05
B-Treg&HiF		3E-14	4E-15	3E-06	3E-06	1E-14	2E-20	1E-02	>0.05	3E-14	3E-16	2E-08	3E-15	2E-04	4E-05	4E-07
C-HiF		8E-11	2E-10	4E-07	3E-08	1E-08	7E-15	>0.05	>0.05	>0.05	>0.05	>0.05	>0.05	>0.05	>0.05	>0.05

Agilent Dataset		Normalized enrichment of gene or gene basket														
Cohort	Pop %	SLC2A1	SLC2A3	VEGFA	VLDLR	ADM	HiF	FOXP3	CTLA4	GITR	RORC	GATA3	Treg	MG	MG Inflam	MG ↑↑NF-kB
A-Treg	24.9	1.17	0.93	0.71	1.12	0.61	0.80	1.12	1.10	1.35	1.17	1.23	1.76	0.82	1.17	0.90
B-Treg&HiF	26.9	1.81	2.19	2.56	2.21	3.11	2.80	1.10	1.04	1.29	1.15	1.19	1.72	0.87	1.13	1.62
C-HiF	21.9	1.77	1.96	3.50	2.14	3.06	3.05	1.01	0.96	1.12	1.01	1.04	1.07	0.80	1.11	1.24
D-Neither	26.4	1.0	1.0	1.0	1.0	1.0	1.0	1.0	1.0	1.0	1.0	1.0	1.0	1.0	1.0	1.0
Cohort		*p*=value of stratified group with respect to 'neither'														
A-Treg		>0.05	>0.05	>0.05	>0.05	>0.05	>0.05	1E-09	5E-04	6E-07	5E-08	1E-07	4E-10	>0.05	>0.05	>0.05
B-Treg&HiF		3E-06	9E-06	1E-03	3E-03	3E-04	8E-07	8E-06	3E-02	1E-08	6E-09	1E-07	1E-11	>0.05	>0.05	>0.05
C-HiF		3E-05	3E-05	4E-05	4E-02	1E-03	1E-06	>0.05	>0.05	>0.05	>0.05	>0.05	>0.05	1E-02	>0.05	>0.05

Gravendeel Dataset		Normalized enrichment of gene or gene basket														
Cohort	Pop %	SLC2A1	SLC2A3	VEGFA	VLDLR	ADM	HiF	FOXP3	CTLA4	GITR	RORC	GATA3	Treg	MG	MG Inflam	MG ↑↑NF-kB
A-Treg	23.0	1.03	0.97	1.01	1.05	0.96	1.09	1.12	1.07	1.07	1.07	1.05	2.72	0.99	1.02	1.02
B-Treg&HiF	32.8	1.21	1.19	1.30	1.16	1.20	2.13	1.16	1.17	1.13	1.08	1.18	4.34	1.03	1.05	1.03
C-HiF	23.8	1.14	1.16	1.25	1.10	1.23	1.95	1.03	1.01	0.99	1.00	0.99	1.10	0.94	1.03	1.06
D-Neither	20.5	1.0	1.0	1.0	1.0	1.0	1.0	1.0	1.0	1.0	1.0	1.0	1.0	1.0	1.0	1.0
Cohort		*p*=value of stratified group with respect to 'neither'														
A-Treg		>0.05	>0.05	>0.05	>0.05	>0.05	>0.05	8E-09	3E-04	2E-08	3E-07	1E-04	6E-08	>0.05	>0.05	>0.05
B-Treg&HiF		1E-14	3E-07	2E-11	5E-04	3E-11	2E-13	5E-15	8E-12	2E-11	6E-12	4E-07	1E-13	>0.05	>0.05	>0.05
C-HiF		2E-11	8E-07	3E-09	3E-02	3E-10	4E-11	5E-02	>0.05	>0.05	>0.05	7E-03	>0.05	>0.05	>0.05	>0.05

CGGA Dataset		Normalized enrichment of gene or gene basket														
Cohort	Pop %	SLC2A1	SLC2A3	VEGFA	VLDLR	ADM	HiF	FOXP3	CTLA4	GITR	RORC	GATA3	Treg	MG	MG Inflam	MG ↑↑NF-kB
A-Treg	24.1	1.38	0.98	2.59	1.10	2.24	1.35	1.30	1.48	1.99	1.07	1.45	1.48	1.15	1.35	1.35
B-Treg&HiF	33.2	2.55	2.30	12.00	2.10	6.23	3.30	1.29	1.53	1.69	1.14	1.72	1.60	1.40	1.41	2.80
C-HiF	14.4	2.23	2.11	10.38	1.35	6.30	3.01	0.97	0.96	0.74	0.91	1.01	1.06	0.71	1.28	1.18
D-Neither	28.3	1.0	1.0	1.0	1.0	1.0	1.0	1.0	1.0	1.0	1.0	1.0	1.0	1.0	1.0	1.0
Cohort		*p*=value of stratified group with respect to 'neither'														
A-Treg		>0.05	>0.05	8E-04	>0.05	1E-05	1E-03	>0.05	2E-02	>0.05	>0.05	2E-02	>0.05	>0.05	>0.05	>0.05
B-Treg&HiF		2E-06	6E-05	7E-11	5E-04	1E-09	1E-13	>0.05	4E-02	>0.05	>0.05	7E-03	>0.05	>0.05	3E-03	3E-04
C-HiF		6E-05	2E-03	6E-07	>0.05	8E-10	2E-12	>0.05	5E-02	>0.05	>0.05	>0.05	>0.05	>0.05	7E-03	>0.05

Table S2. Characteristics of stratification of GBM tumors by steroidogenic fractionation, we *p*=values derived from t-tests performed against the asteriodogenic cohort.

U133 Dataset		Normalized enrichment of gene or gene basket																		
Cohort	Pop %	SLC22A11	SLC22A9	SLCO4A1	CYP11A1	CYP17A1	CYP19A1	HSD17B3	HSD3B1	AKR1C1	AKR1C3	MG	MG Inflam	MG ↑↑NF-kB	Androgen	Estrogen	CD33	IL10	EBI3	MNDA
A-T&P, low E	28.5	1.17	1.70	1.47	1.90	1.35	0.94	0.85	1.30	1.37	0.80	0.6	1.4	0.7	2.3	0.9	0.77	0.78	0.92	0.77
B-E&P, low A	25.4	1.10	1.57	1.48	1.83	1.19	2.01	0.84	1.19	1.29	1.13	1.1	1.4	1.2	1.7	1.8	1.65	1.41	1.24	1.28
C-E&A, low P	21.6	1.16	1.73	1.42	2.20	1.35	1.40	0.79	1.20	2.88	2.94	1.0	1.4	1.5	2.2	1.5	1.59	1.24	1.44	1.25
D-Asteroid	24.4	1.0	1.0	1.0	1.0	1.0	1.0	1.0	1.0	1.0	1.0	1.0	1.0	1.0	1.0	1.0	1.0	1.0	1.0	1.0
Cohort		*p*=value of stratified group with respect to asteroidogenic tumor cohort																		
A-T&P, low E		3E-04	2E-06	>0.05	9E-03	1E-06	>0.05	>0.05	1E-04	1E-03	5E-05	>0.05	>0.05	5E-02	6E-10	2E-06	9E-04	>0.05	1E-02	2E-02
B-E&P, low A		>0.05	4E-02	>0.05	5E-03	>0.05	2E-07	>0.05	3E-02	6E-04	1E-05	3E-04	6E-01	9E-05	2E-02	1E-14	1E-11	3E-04	1E-06	2E-09
C-E&A, low P		5E-03	1E-05	>0.05	3E-04	8E-05	3E-09	>0.05	1E-03	>0.05	>0.05	7E-04	1E-02	7E-07	2E-07	2E-22	6E-18	8E-04	7E-09	2E-14

Agilent Dataset		Normalized enrichment of gene or gene basket																		
Cohort	Pop %	SLC22A11	SLC22A9	SLCO4A1	CYP11A1	CYP17A1	CYP19A1	HSD17B3	HSD3B1	AKR1C1	AKR1C3	MG	MG Inflam	MG ↑↑NF-kB	Androgen	Estrogen	CD33	IL10	EBI3	MNDA
A-T&P, low E	28.9	1.08	1.00	1.05	1.18	1.06	1.15	1.04	1.01	1.28	1.43	0.94	0.99	0.98	1.50	1.16	1.50	1.11	1.10	2.14
B-E&P, low A	25.4	1.04	0.86	1.07	1.12	1.02	1.39	1.23	1.01	1.17	1.75	1.21	0.92	1.07	1.00	1.53	1.43	1.20	1.02	1.86
C-E&A, low P	26.4	1.08	0.71	1.01	1.17	1.08	1.77	1.10	1.03	1.48	2.17	1.23	1.08	1.39	1.58	1.91	1.36	1.17	1.07	1.92
D-Asteroid	19.4	1.0	1.0	1.0	1.0	1.0	1.0	1.0	1.0	1.0	1.0	1.0	1.0	1.0	1.0	1.0	1.0	1.0	1.0	1.0
Cohort		*p*=value of stratified group with respect to asteroidogenic tumor cohort																		
A-T&P, low E		1E-03	>0.05	>0.05	2E-02	9E-04	>0.05	>0.05	>0.05	>0.05	>0.05	>0.05	>0.05	>0.05	2E-12	>0.05	>0.05	>0.05	>0.05	>0.05
B-E&P, low A		>0.05	>0.05	>0.05	4E-02	>0.05	>0.05	6E-03	>0.05	>0.05	>0.05	>0.05	>0.05	>0.05	>0.05	>0.05	>0.05	>0.05	>0.05	>0.05
C-E&A, low P		4E-02	1E-03	>0.05	>0.05	3E-02	>0.05	>0.05	2E-02	>0.05	>0.05	7E-03	>0.05	>0.05	5E-05	1E-02	5E-02	>0.05	2E-02	>0.05

Gravendeel Dataset		Normalized enrichment of gene or gene basket																		
Cohort	Pop %	SLC22A11	SLC22A9	SLCO4A1	CYP11A1	CYP17A1	CYP19A1	HSD17B3	HSD3B1	AKR1C1	AKR1C3	MG	MG Inflam	MG ↑↑NF-kB	Androgen	Estrogen	CD33	IL10	EBI3	MNDA

A-T&P, low E	26.2	1.07	1.11	1.03	1.03	1.05	1.08	1.06	1.06	1.02	1.01	1.06	0.96	1.03	2.09	1.12	1.01	1.01	1.03	1.00
B-E&P, low A	21.3	1.04	1.06	1.03	1.03	1.02	1.04	1.02	1.03	1.01	1.01	1.04	0.95	1.05	1.13	2.06	1.16	1.06	1.10	1.21
C-E&A, low P	26.2	1.23	1.23	1.13	1.21	1.18	1.22	1.17	1.22	1.14	0.99	1.09	0.96	1.01	3.26	2.34	1.20	1.14	1.16	1.21
D-Asteroid	26.2	1.0	1.0	1.0	1.0	1.0	1.0	1.0	1.0	1.0	1.0	1.0	1.0	1.0	1.0	1.0	1.0	1.0	1.0	1.0
Cohort		*p*=value of stratified group with respect to asteroidogenic tumor cohort																		
A-T&P, low E		7E-04	2E-07	>0.05	>0.05	3E-04	3E-06	7E-02	1E-05	>0.05	>0.05	>0.05	>0.05	>0.05	4E-15	3E-06	>0.05	1E-02	>0.05	>0.05
B-E&P, low A		>0.05	2E-02	>0.05	>0.05	>0.05	6E-03	>0.05	>0.05	>0.05	>0.05	>0.05	>0.05	>0.05	>0.05	6E-03	3E-12	1E-04	2E-04	5E-12
C-E&A, low P		5E-20	4E-14	9E-08	1E-04	2E-24	1E-24	9E-06	5E-30	9E-15	>0.05	2E-02	>0.05	>0.05	3E-27	1E-24	5E-12	3E-10	3E-08	1E-10

CGGA Dataset		Normalized enrichment of gene or gene basket																		
Cohort	Pop %	SLC22A11	SLC22A9	SLCO4A1	CYP11A1	CYP17A1	CYP19A1	HSD17B3	HSD3B1	AKR1C1	AKR1C3	MG	MG Inflam	MG ↑↑NF-kB	Androgen	Estrogen	CD33	IL10	EBI3	MNDA
A-T&P, low E	23.5	1.05		1.76	1.33	1.19	1.20	4.80		2.11	3.50	1.94	0.93	0.48		1.18	1.71	3.12	1.04	1.91
B-E&P, low A	25.1	1.13		2.81	1.76	1.07	3.67	4.60		1.96	4.51	2.91	1.06	1.39		3.13	3.36	4.25	1.71	4.39
C-E&A, low P	26.2	1.19		2.50	2.67	1.15	2.48	3.41		5.19	7.32	3.55	0.99	1.59		4.01	3.49	5.17	2.16	5.55
D-Asteroid	25.1	1.0		1.0	1.0	1.0	1.0	1.0		1.0	1.0	1.0	1.0	1.0		1.0	1.0	1.0	1.0	1.0
Cohort		*p*=value of stratified group with respect to asteroidogenic tumor cohort																		
A-T&P, low E		>0.05		4E-02	1E-03	>0.05	>0.05	6E-07		>0.05	2E-02	3E-02	>0.05	1E-02		3E-02	>0.05	1E-02	>0.05	>0.05
B-E&P, low A		1E-02		2E-04	1E-03	>0.05	6E-11	6E-04		>0.05	9E-03	5E-05	>0.05	>0.05		5E-05	6E-05	3E-04	>0.05	3E-03
C-E&A, low P		3E-02		2E-04	4E-05	>0.05	6E-05	3E-06		1E-04	1E-07	2E-08	>0.05	>0.05		2E-08	2E-05	2E-06	6E-02	1E-05
